# Happiness in US military veterans: Results from a nationally representative study

**DOI:** 10.1371/journal.pone.0313609

**Published:** 2024-12-11

**Authors:** Hun Kang, Ian C. Fischer, Peter J. Na, Robert H. Pietrzak

**Affiliations:** 1 Department of Social and Behavioral Sciences, Yale School of Public Health, New Haven, CT, United States of America; 2 U.S. Department of Veterans Affairs National Center for Posttraumatic Stress Disorder, VA Connecticut Healthcare System, West Haven, CT, United States of America; 3 Department of Psychiatry, Yale School of Medicine, New Haven, CT, United States of America; 4 VA Connecticut Healthcare System, West Haven, CT, United States of America; University of Foggia: Universita degli Studi di Foggia, ITALY

## Abstract

In line with the US Department of Veterans Affairs’ adoption of a *Whole Health* approach to healthcare, there has been growing interest in factors linked to veterans’ perceptions of well-being. To date, no known study has examined levels and correlates of perceived happiness in this population. To examine this question, we analyzed data from the National Health and Resilience in Veterans Study, which surveyed a nationally representative sample of 4,069 US military veterans. Overall, veterans reported mean happiness scores of 5.41 out of 7. Greater purpose in life was the strongest correlate of happiness, followed by lower severity of depressive symptoms, and higher optimism, emotional stability, and resilience. Among veterans who screened positive for depression, those who scored higher on measures of optimism, emotional stability, and resilience reported greater happiness. Interventions to leverage these modifiable psychosocial characteristics may help promote happiness and subjective well-being in this population.

## Background

The U.S. Department of Veterans Affairs has begun to shift from a disease-oriented healthcare model to one that prioritizes subjective well-being [1]. This new model, referred to as *Whole Health*, is defined as “an approach to care that empowers and equips a person to take charge of their health and well-being and live their life to the fullest” [1]. Accordingly, understanding subjective perceptions of health and well-being (i.e., “*What matters to you*?” instead of “*What is the matter with you*?”) is critical to health promotion [2].

Happiness, one of the core components of subjective well-being [3], has been linked to health behaviors such as physical activity and healthy eating [4], better physical and mental health outcomes [5–7], and lower mortality risk [8]. To date, while several studies examined related psychological constructs such as quality of life and social well-being among veterans [9, 10], no known study has examined levels and correlates of perceived happiness in this population, which can help inform more person-centered, preventative healthcare approaches.

In the current study, we analyzed data from the National Health and Resilience in Veterans (NHRVS), which surveyed a nationally representative sample of US veterans, to (1) examine levels of happiness; (2) identify and quantify key correlates of happiness; and (3) characterize psychosocial factors that moderate strong negative correlates of happiness.

## Methods and materials

### Sample

A total of 4,069 veterans participated in the NHRVS. The NHRVS sample was drawn from KnowledgePanel, a research panel of more than 50,000 U.S. households maintained by Ipsos, a survey research firm. KnowledgePanel is a probability-based, online, non-volunteer access survey panel of a nationally representative sample of U.S. veterans that covers approximately 98% of U.S. households. Panel members are recruited through national random samples, originally by telephone and now almost entirely by postal mail. KnowledgePanel recruitment uses dual sampling frames that include both listed and unlisted telephone numbers, telephone and non-telephone households, and cell-phone-only households, as well as households with and without Internet access.

To permit generalizability of study results to the entire population of U.S. veterans, the Ipsos statistical team computed poststratification weights using the following benchmark distributions of U.S. military veterans from the most contemporaneous (August 2019) Current Veteran Population Supplemental Survey of the U.S. Census Bureau’s American Community Survey: age, gender, race/ethnicity, Census Region, metropolitan status, education, household income, branch of service, and years in service. An iterative proportional fitting (raking) procedure was used to produce the final poststratification weights.

### Ethics statement

All participants provided electronic informed consent. This study was approved by the Human Subjects Committee of the Veterans Affairs Connecticut Healthcare System.

### Assessments

Happiness was assessed with a single, 7-point item from the Subjective Happiness Scale (SHS; [11]), which asks “*In general*, *I consider myself*:” “Not a very happy person” (1) to “A very happy person” (7).

A broad range of sociodemographic, military, health, personality, and psychosocial characteristics was assessed in addition to the assessment of subjective happiness. Sociodemographic characteristics included age, sex, race/ethnicity, education level, marital status, employment status, and household income. Military characteristics included combat veteran status, years in military, and perceptions of the extent to the military had a positive vs. negative effect on one’s life. Health characteristics included physical health difficulties (a composite score of number of medical conditions, somatic symptoms, any disability in activities of daily living, and any disability in instrumental activities of daily living), physical exercise, adverse childhood experiences, cumulative trauma burden, and current psychological distress (a composite score of major depressive disorder symptoms, generalized anxiety disorder symptoms, and posttraumatic stress disorder symptoms). Personality characteristics included extraversion, agreeableness, emotional stability, conscientiousness, and openness to experiences. Psychosocial characteristics included protective psychosocial characteristics (a composite score of resilience, purpose in life, dispositional optimism, dispositional gratitude, curiosity, grit, and community integration), social connectedness (a composite score of structural social support, perceived social support, secure attachment), religiosity/spirituality (a composite score of religious service attendance, private spiritual activities, and intrinsic religiosity), and altruism (a composite score of altruistic behavior and provision of social support). Please see S1 Table for further details.

### Data analysis

First, we conducted bivariate correlations between happiness scores and a broad range of characteristics. Second, we performed a multiple linear regression to identify independent correlates of happiness scores; variables correlated with happiness at *p*<0.05 level in bivariate correlations were entered into the regression. When multi-component variables were identified as significant correlates of happiness scores, we conducted planned post-hoc analyses to examine specific components significantly associated with this measure. Third, we conducted a relative importance analysis (RIA) to examine the relative variance explained (RVE) by each significant correlate; this analysis identified the proportion of variance in happiness scores explained by each independent variable while accounting for intercorrelations among these variables [12]. Fourth, we explored interactions between the strongest negative and positive correlates of happiness scores. Statistical analyses were performed with SPSS version 25 and R Studio, and data were visualized using Microsoft Excel.

## Results

The mean level of happiness in veterans was 5.41 (SD = 1.40; range = 1–7).

Table 1 summarizes sample characteristics and results of analyses. Results of a multiple linear regression revealed that happiness scores were positively associated with older age, combat veteran status, greater perceptions of the military having a positive effect on one’s life, extraversion, agreeableness, emotional stability, protective psychosocial characteristics (post-hoc analysis: purpose in life, β = 0.33, *p*<0.001; optimism, β = 0.11, *p*<0.001; resilience, β = 0.06, *p*<0.001; curiosity, β = 0.04, *p* = 0.001; gratitude, β = 0.04, *p* = 0.002), and social connectedness (post-hoc analysis: received social support, β = 0.08; secure attachment, β = 0.06, both *p*’s<0.001), and negatively associated with White, non-Hispanic race/ethnicity, college graduate or higher education, adverse childhood experiences, cumulative trauma burden, current psychological distress (post-hoc analysis: current major depressive disorder (MDD) symptoms, β = -0.16, *p*<0.001), and conscientiousness.

**Table 1:** Sample characteristics and results of bivariate correlation and multiple regression analyses predicting happiness scores (n = 4,069).

	Sample characteristics	Bivariate correlation with happiness scores	Multiple regression analysis (R^2^ = 0.64)	
	Weighted mean (SD) or unweighted *n* (Weighted %)	*r*	β	*t*
*Sociodemographic characteristics*				
Age	62.2 (15.7)	0.31**	0.07**	4.47
Male sex^1^	3,564 (90.2%)	0.10**	0.00	0.35
White, non-Hispanic race/ethnicity^2^	3,318 (78.1%)	-0.04*	-0.05**	4.45
College graduate or higher education^3^	1,827 (32.7%)	0.04*	-0.05**	5.09
Married or partnered^4^	2,885 (72.4%)	0.10**	0.01	0.59
Retired^5^	2,225 (44.3%)	0.17**	0.02	1.84
Household income $60k or higher^6^	2,357 (58.5%)	0.10**	-0.01	0.56
*Military characteristics*				
Combat veteran^7^	1,353 (34.9%)	-0.04*	0.02*	2.23
Years in military	4.4 (1.9)	-0.01	-	-
Positive effect of military on life	2.0 (1.4)	0.25**	0.02*	2.22
*Health characteristics*				
Physical health difficulties	0.0 (1.0)	-0.22**	-0.02	1.70
Physical exercise	34.0 (40.5)	0.04**	0.00	0.37
Adverse childhood experiences	1.5 (2.0)	-0.29**	-0.02*	2.12
Cumulative trauma burden	8.9 (8.5)	-0.17**	-0.03*	2.35
Current psychological distress	0.0 (1.0)	-0.51**	-0.15**	10.45
*Personality*				
Extraversion	3.8 (1.5)	0.37**	0.07**	5.97
Agreeableness	5.0 (1.2)	0.38**	0.05**	3.93
Emotional stability	5.2 (1.4)	0.53**	0.09**	6.65
Conscientiousness	5.7 (1.2)	0.35**	-0.04**	3.47
Openness to experiences	4.8 (1.2)	0.26**	-0.01	-0.76
*Psychosocial factors*				
Protective psychosocial characteristics	0.0 (1.0)	0.69**	0.49**	31.70
Social connectedness	0.0 (1.0)	0.55**	0.12**	9.35
Religiosity/spirituality	0.0 (1.0)	0.22**	-0.01	1.22
Altruism	0.0 (1.0)	0.30**	0.00	0.21

*Note*. SD = standard deviation.

Overall model: F(df = 23, 3874) = 296.98, *p*<0.001.

Significant association

*p<0.05

**p<0.01.

^1^Reference group: Female sex

^2^Reference group: Non-white/Hispanic race/ethnicity (Black/Non-Hispanic, Other/Non-Hispanic, 2+ Races/Non-Hispanic, or Hispanic)

^3^Reference group: Less than college graduate level of education

^4^Reference group: Not married/partnered (widowed, divorced, separated, or never married)

^5^Reference group: Employed or not working for other reasons (e.g., disability)

^6^Reference group: Household income less than $60k

^7^Reference group: No service history in a combat or war zone

**Fig 1:**
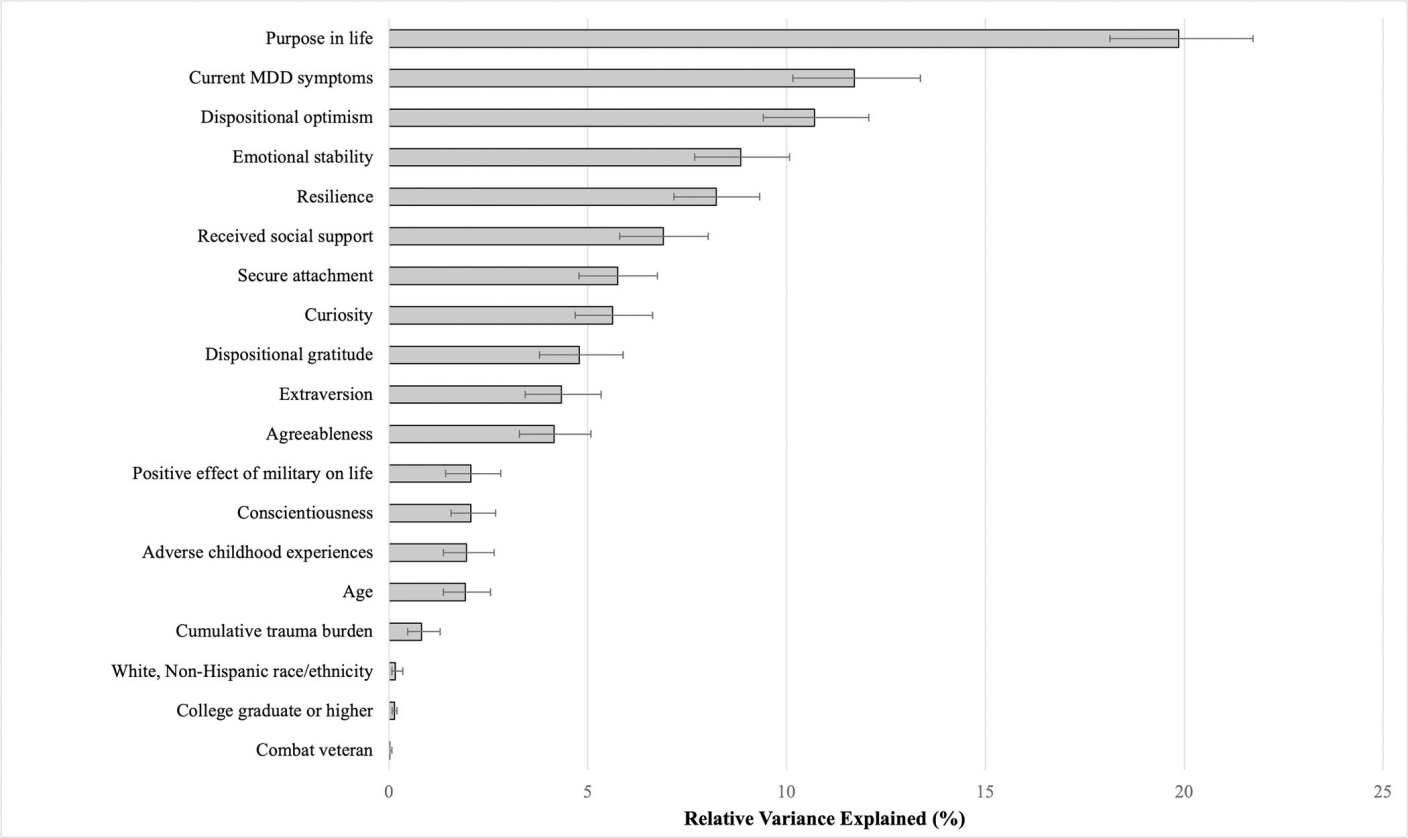
Results of relative importance analysis of factors associated with happiness scores in U.S. military veterans. MDD = major depressive disorder. Error bars represent 95% confidence intervals.

RIA results revealed that the majority (>75%) of explained variance in happiness scores was accounted for by higher purpose life (19.9% RVE), lower MDD symptoms (11.7%), higher optimism (10.7%), emotional stability (8.8%), resilience (8.2%), received social support (6.9%), secure attachment style (5.8%), and curiosity (5.6%; Fig 1).

**Fig 2:**
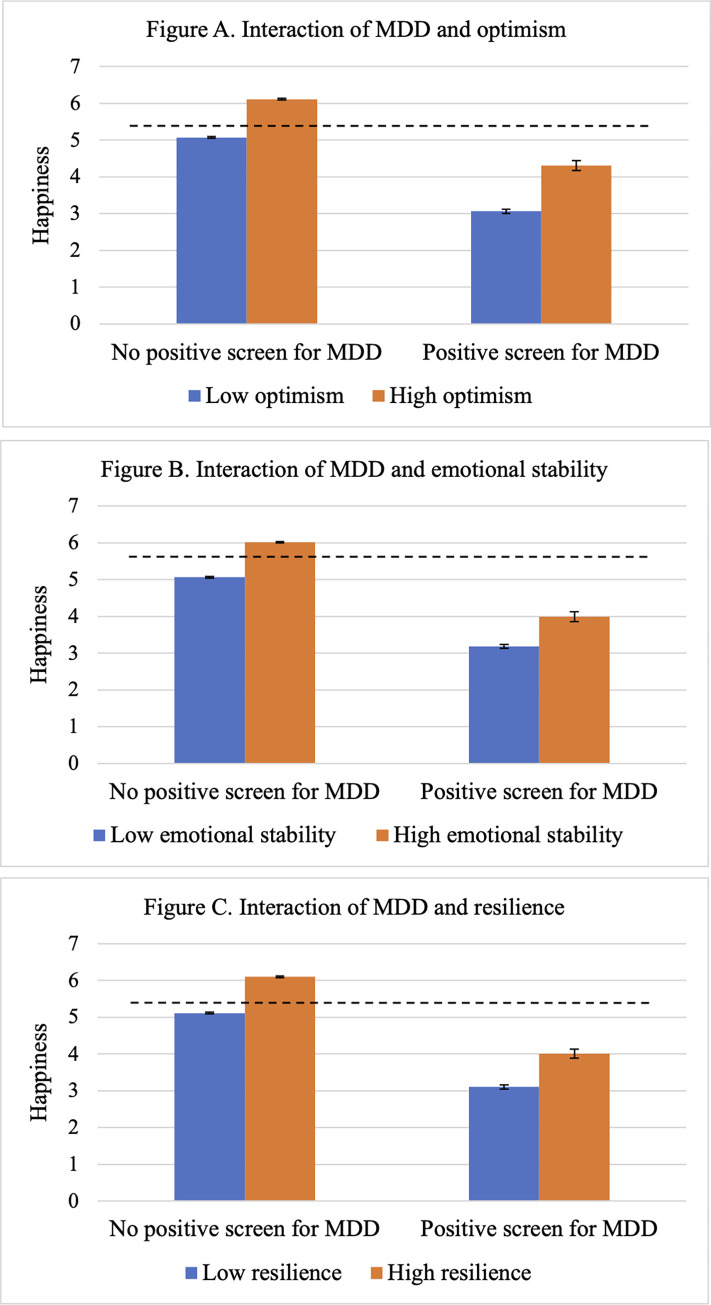
Results of interaction analyses. MDD = major depressive disorder. Error bars represent 95% confidence intervals. Dashed line represents mean happiness score in the full sample.

As shown in Fig 2, significant interactions were observed between MDD symptoms and optimism (F(3, 4024) = 1285.95; Fig 2A); emotional stability (F(3, 4016) = 1027.70; Fig 2B); and resilience (F(3, 4017) = 1185.87; Fig 2C), all *p*’s<0.001. Specifically, among veterans who screened positive for MDD, veterans with higher levels of optimism (Cohen’s *d* = 1.10, 95%CI = 0.80–1.41), emotional stability (*d* = 0.80, 95%CI = 0.49–1.12), and resilience (*d* = 0.51, 95%CI = 0.25–0.78) had higher happiness scores.

## Discussion

To our knowledge, this is the first study to examine the level and correlates of happiness in a nationally representative sample of US veterans. On average, veterans reported mean happiness scores of 5.41/7, which while lower than mean SHS scores observed in community-based US adult (mean = 5.62, *t* = 2.19, *p* = 0.03) and retired US adult (mean = 5.62, *t* = 3.79, *p*<0.001) samples [11], were small magnitude differences (both *d*’s = 0.17).

Purpose in life was the strongest correlate of happiness, consistent with prior work demonstrating its associations with mental health outcomes in veterans [13, 14]. Given its link to adaptive cognitive and behavioral processes (e.g., psychological flexibility; [15]), interventions to promote purpose in life [16] may help increase happiness in veterans. Such interventions also resonate with VA’s Whole Health initiative, which places an emphasis on purpose in life in enhancing well-being [1].

Among veterans who screened positive for MDD, those who were more optimistic, emotionally stable, and resilient reported higher levels of happiness, despite their scoring lower relative to veterans without MDD. These adaptive characteristics may promote happiness by facilitating adaptive coping strategies such as positive reframing [17]; fostering positive emotions and buffering stress [18]; and lowering emotional reactivity to stressors [19], even among veterans with clinically significant MDD symptoms. While interventions leveraging these factors [20–22] may help promote happiness in veterans, further research is needed to evaluate their effectiveness among veterans with MDD.

Limitations of this study include the cross-sectional design, use of a single-item measure of happiness, and reliance on self-report measures. Further studies that employ longitudinal and qualitative study designs [23, 24] and employ multi-dimensional measures of happiness [11, 25] are needed to provide a richer understanding on how participants perceive and experience happiness.

Despite these limitations, results provide preliminary insight into potential directions for Whole Health interventions designed to promote happiness and subjective well-being in veterans. Further research is needed to replicate these findings in other veteran samples; evaluate dimensions of happiness and other aspects of well-being (e.g., hedonic vs. eudemonic well-being [26]) evaluate the effectiveness of prevention and treatment efforts in targeting modifiable factors such as purpose in life in promoting happiness and overall well-being in this population.

## Supporting information

S1 Table Assessments of sociodemographic, military, health, personality, psychosocial characteristics. (DOCX)
