# Supplementary material for: Happiness in US military veterans: Results from a nationally representative study
Source: PLoS One. 2024 Dec 11;19(12):e0313609. doi: 10.1371/journal.pone.0313609 (PMC11633995; doi:10.1371/journal.pone.0313609)
Supplement: S1 Table — (DOCX) [file pone.0313609.s001.docx]

S1 Table. Assessments of sociodemographic, military, health, personality, psychosocial characteristics

| **Measure** | **Assessment** | **Factor loading** |
| --- | --- | --- |
| *Sociodemographic characteristics* | Age, sex, race/ethnicity, education, marital status, employment status, household income | - |
|  |  |  |
| *Military characteristics* |  |  |
| Combat veteran | Did you ever serve in a combat or war zone? | - |
| Years in military | How many years did you spend in the military? | - |
| Positive effect of military on life | How has being in the military affected your life? Score range: 1-7 (1=Strong negative effect; 7=Strong positive effect) | - |
|  |  |  |
| *Health characteristics* |  |  |
| Physical health difficulties |  |  |
| Number of medical conditions | Sum of number of medical conditions endorsed in response to question: “Has a doctor or healthcare professional ever told you that you have any of the following medical conditions?” (e.g., arthritis, cancer, diabetes, heart disease, asthma, kidney disease). Range: 0-24 conditions. | 0.673 |
| Somatic symptoms | Score on Somatization subscale of the Brief Symptom Inventory-18.^1^ | 0.761 |
| ADL disability | Any disability in activities of daily living. The following question was asked: “At the present time, do you need help from another person to do the following?” (e.g., bathe; walk around your home or apartment; get in and out of chair). Endorsement of any of these activities was indicative of having a disability with an activity of daily living.^2^ | 0.681 |
| IADL disability | Any disability in instrumental activities of daily living. The following question was asked: “At the present time, do you need help from another person to do the following?” (e.g., pay bills or manage money; prepare bills; get dressed). Endorsement of any of these activities was indicative of having a disability with an instrumental activity of daily living. | 0.744 |
| Physical exercise | Score on Godin Leisure-Time Exercise Questionnaire.^3^ | - |
| Adverse childhood experiences | Score on Adverse Childhood Experiences Questionnaire.^4^ | - |
| Cumulative trauma burden | Count of potentially traumatic events on the Life Events Checklist for DSM-5.^5^ | - |
| Current psychological distress |  |  |
| MDD symptoms | Score on the Patient Health Questionnaire-2.^6^ | 0.896 |
| GAD symptoms | Score on the Generalized Anxiety Disorder-2.^6^ | 0.892 |
| PTSD symptoms | Score on past-month version of the PTSD Checklist for DSM-5.^7^ | 0.816 |
|  |  |  |
| *Personality characteristics* |  |  |
| Extraversion | Score on Extraversion subscale of the Ten-Item Personality Inventory.^8^ | - |
| Agreeableness | Score on Agreeableness subscale of the Ten-Item Personality Inventory.^8^ | - |
| Emotional stability | Score on Conscientiousness subscale of the Ten-Item Personality Inventory.^8^ | - |
| Conscientiousness | Score on Emotional Stability subscale of the Ten-Item Personality Inventory.^8^ | - |
| Openness to experiences | Score on Openness to Experiences subscale of the Ten-Item Personality Inventory.^8^ | - |
|  |  |  |
| *Psychosocial characteristics* |  |  |
| Protective psychosocial characteristics |  |  |
| Resilience | Score on Connor-Davidson Resilience Scale-10.^9^ | 0.770 |
| Purpose in life | Score on Purpose in Life Test-Short Form.^10^ | 0.834 |
| Dispositional optimism | Score on single-item measure of optimism from Life Orientation Test-Revised^11^; “In uncertain times, I usually expect the best”); rating 1=strongly disagree to 7=strongly agree. | 0.752 |
| Dispositional gratitude | Score on single-item measure of gratitude from Gratitude Questionnaire^12^: “I have so much in life to be thankful for” (rating 1=strongly disagree to 7=strongly agree). | 0.637 |
| Curiosity/exploration | Score on single-item measure of curiosity/exploration from Curiosity and Exploration Inventory-II^13^; “I frequently find myself looking for new opportunities to grow as a person (e.g., information, people, resources”) rating 1=strongly disagree to 7=strongly agree. | 0.722 |
| Grit | Score on Short Grit Scale.^14^ | 0.623 |
| Community integration | Perceived level of community integration: “I feel well integrated in my community (e.g., regularly participate in community activities)” rating 1=strongly disagree to 7=strongly agree. | 0.647 |
| Social connectedness |  |  |
| Structural social support | Response to question: “About how many close friends and relatives do you have (people you feel at ease with and can talk to about what is on your mind)?” | 0.636 |
| Perceived social support | Score on 5-item version of the Medical Outcomes Study Social Support Scale.^15,16^ | 0.804 |
| Attachment style | Endorsement of secure attachment (response a) to the following question: “Please select the statement below that best describes your feelings and attitudes in relationships^17^: (a) feeling that it is easy to get close to others and feeling comfortable with them (secure); (b) feeling uncomfortable being close to others (avoidant); or (c) feeling that others are reluctant to get close (anxious/ambivalent). | 0.783 |
| Religiosity/spirituality |  |  |
| Religious service attendance | Frequency of attending religious services on Duke University Religion Index.^18^ | 0.866 |
| Private spiritual activities | Frequency of private spiritual activities on Duke University Religion Index.^18^ | 0.870 |
| Intrinsic religiosity | Score on measure of intrinsic religiosity on Duke University Religion Index^18^; sample item: “In my life, I experience the presence of the Divine (i.e., God).” | 0.878 |
| Altruism |  |  |
| Altruistic behavior | Frequency of engagement in helping others with instrumental activities of daily living^19^: “How often have you helped a friend, neighbor, or relative other than your spouse or partner with errands, child care, housework, transportation, or other tasks in the PAST YEAR?” (Response options: Never, 1 to 10 times, 11-50 times, 51-99 times, and 100 or more times). | 0.767 |
| Provision of social support | Score on modified 5-item version of the Medical Outcomes Study Social Support Scale^15,16^ that assesses the extent to which an individual provided support to others (e.g., “How often do you provide the following kinds of support to others who need it? – I am someone that helps others with daily chores if they were sick.” | 0.767 |

**References**

1. Derogatis LR: Brief Symptom Inventory 18 (BSI-18). Administration, Scoring and Procedures Manual, Minneapolis, MN, NCS Pearson, Inc., 2001.
2. Hardy SE, Gill TM: Recovery from disability among community-dwelling older persons. JAMA 2004; 291:1596-1602.
3. Godin G: The Godin-Shephard leisure-time physical activity questionnaire. Health & Fitness Journal of Canada 2011; 4:18-22.
4. Felitti VJ, Anda RF, Nordenberg D, et al: Relationship of childhood abuse and household dysfunction to many of the leading causes of death in adults. The Adverse Childhood Experiences (ACE) Study. Am J Prev Med 1998; 14:245-258
5. Weathers FW, Blake DD, Schnurr PP, et al.: The Life Events Checklist for DSM-5 (LEC-5). 2019. Instrument available from the National Center for PTSD at [www.ptsd.va.gov](http://www.ptsd.va.gov).
6. Kroenke K, Spitzer RL, Williams JB, et al: An ultra-brief screening scale for anxiety and depression: the PHQ-4. Psychosomatics 2009; 50:613-621.
7. Weathers FW, Litz BT, Keane TM, Palmieri PA, Marx BP, Schnurr PP. The PTSD Checklist *for*DSM-5 (PCL-5). 2013. Scale available from the National Center for PTSD at [www.ptsd.va.gov](https://www.ptsd.va.gov/).
8. Gosling SD, Rentfrow PJ, Swann WB: A very brief measure of the big-five personality domains. J Res Pers 2003; 37:504e528.
9. Campbell-Sills L, Stein MB: Psychometric analysis and refinement of the Connor-Davidson Resilience Scale (CD-RISC): validation of a 10-item measure of resilience. J Trauma Stress 2007; 20:1019-1028.
10. Schulenberg SE, Schnetzer LW, Buchanan EM: The Purpose in Life Test-Short Form: development and psychometric support. J Happiness Stud 2010; 20:1-16.
11. Scheier MF, Carver CS, Bridges MW: Distinguishing optimism from neuroticism (and trait anxiety, self-mastery, and self-esteem): a re-evaluation of the Life Orientation Test. J Pers Soc Psychol 1994; 67:1063-1078.
12. McCullough ME, Emmons RA, Tsang J: The grateful disposition: a conceptual and empirical topography. J Pers Soc Psychol 2002; 82:112-127.
13. Kashdan TB, Gallagher MW, Silvia PJ, et al: The Curiosity and Exploration Inventory-II: development, factor structure, and initial psychometrics. J Res Pers 2009: 43:987-998.
14. Duckworth AL,Quinn PD: Development and validation of the short grit scale (grit-s). J Pers Assess 2009; 91:166-174.
15. Sherbourne CD, Stewart, AL: The MOS social support survey. Soc Sci Med 1991; 32: 705-714.
16. Amstadter AB, Begle AM, Cisler JM, et al: Prevalence and correlates of poor self-rated health in the United States: the national elder mistreatment study. Am J Geriatr Psychiatry 2010; 18:615-623.
17. Hazan C, Shaver, PR: Love and work: An attachment-theoretical perspective. J Pers Soc Psychol 1990; 59:270-280.
18. Koenig HG, Büssing A: The Duke University Religion Index (DUREL): A five-item measure for use in epidemiological studies. Religions 2010; 1:78-85.
19. Brown SL, Nesse RM, Vinokur AD, et al: Providing social support may be more beneficial than receiving it: results from a prospective study of mortality. Psychol Sci 2003; 14:320-32
